# Profile of dog bite injuries in patients presenting at Kimberley Hospital Complex’s emergency and gateway centres, 2015 to 2017

**DOI:** 10.4102/phcfm.v12i1.2301

**Published:** 2020-05-21

**Authors:** Nyitiba Ishaya, Talat Habib, Cornel van Rooyen, Wilhelm J. Steinberg

**Affiliations:** 1 Department of Family Medicine, School of Clinical Medicine, Faculty of Health Sciences, University of the Free State, Bloemfontein, South Africa; 2 Department of Biostatistics, School of Clinical Medicine, Faculty of Health Sciences, University of the Free State, Bloemfontein, South Africa

**Keywords:** prevalence, dog bite injury, profile, notification, Kimberley, Northern Cape

## Abstract

**Background:**

Dog bite injuries in humans remain a public health problem. There is limited nationally representative data on the magnitude of the problem and the epidemiological profile of dog bite injuries in South Africa.

**Aim:**

To describe the profile of dog bite injuries in patients presenting to Kimberley Hospital Complex (KHC) emergency and gateway centres. To determine the prevalence of dog bite injuries amongst all patients presenting to these centres and the compliance of mandatory notification of dog bites.

**Setting:**

Kimberley Hospital Complex emergency and gateway centres.

**Methods:**

A retrospective review of all dog bite cases who presented to KHC from August 2015 to July 2017. The total number of all patients who presented were taken into consideration for calculating the prevalence of dog bite cases.

**Results:**

During the study period, 433 dog bite cases were identified out of 107 731 patients seen at emergency and gateway centres, giving a prevalence of 0.4%. Of all cases, 62.4% were male patients and 37.6% were female patients. Most affected age group was between 10 and 19 years (19.6%). Category II exposure type accounted for the majority of the cases (59.4%). Unvaccinated dogs were incriminated in 61.9% of cases. Stray dogs were responsible for 83.1% of all injuries. More than half of the cases (47.9%) were notified by the treating doctors.

**Conclusion:**

Dog bite injuries in Kimberley were commonest in children and adolescents. The prevalence tended to decrease in adulthood with advancing age groups. Most bites resulted from unvaccinated stray dogs. Only about half of the cases were notified to the appropriate authorities. Prevalence of dog bite injuries amongst patients presenting at KHC resulted in the low rate of 0.4%. Awareness needs to be created amongst health care providers on the importance of notification of all exposure to rabies. More efforts are required at the prevention of dog bites in children and adolescents through stringent measures to limit the number of free-roaming dogs.

## Introduction

The close association between humans and dogs began many centuries ago.^1^ The dog used to be a wild animal domesticated by man but did not completely lose its wild animal instincts, including behaviours that may lead to attacks on humans. Dog bite injuries in humans remain a public health problem as it is placed worldwide amongst the top 12 causes of non-fatal injuries.^2^ It poses a major public health threat in both developing and developed nations. Whilst there are no global estimates of dog bite prevalence, studies suggest that dog bite injuries account for tens of millions of injuries annually and is important because of its potential risk of rabies.^3^ In low- and middle-income countries, epidemiology is rather fragmented with children amongst the high-risk groups.^2^

In South Africa, rabies is a notifiable disease according to Regulation 328 of *Health Act* 63 of 1977 (*National Health Act,* No. 61 of 2003).^4,5,6^ Therefore, incidents of human exposure to infections (dog, mongoose, bats and cat scratches), frank cases and deaths from rabies are notified by the treating health care practitioner to the local or provincial Department of Health via the Disease Surveillance Unit.^7^ A detailed history of the exposure, information on the inflicting animal (where possible), type of bite (classification of wound), pre-exposure treatment given and time of presentation of the case are recorded.^7^ However, anecdotal evidence shows doctors are reluctant to report dog bite injuries. Because of the high crime rate in South Africa, large breed dogs are popular and many dogs are taught to be aggressive with the intention of combating crimes.^8^ Demography of companion animals in South Africa indicated 3.93 million dogs in 1992.^9^ Currently, South Africa has a dog population of 9.1 million.^9^ Whilst there are limited data on the owned dog and free-roaming dog population in South Africa, a study in Mpumalanga, South Africa, showed little evidence of free-roaming dogs.^10^

Dog bites are the second most common injury sustained by humans from animals after snake bites.^10^ It accounts for about 1.5% of all trauma emergency presentations at the Red Cross War Memorial Children’s Hospital in Cape Town over a 13.5-year period.^11^ South African and other international studies have shown that children younger than 7 years are more prone to be bitten.^11^ Most affected areas are the head and neck, possibly because their height put them at the same level with the dog.^12^ Dog breeds associated with more aggressive behaviour are Pitbull terriers, Rottweilers, German shepherds and Dobermans.^11^

Dog bite injuries are divided into three categories of exposure to rabies:^13^

- Category I: Touching, feeding of animals or licks on intact skin.
- Category II: Nibbling of uncovered skin, minor scratches or abrasions without bleeding.
- Category III: Single or multiple transdermal bites or scratches, licks on broken skin or contamination of mucous membrane with saliva licks.

For category I, no treatment is required, whereas for category II immediate vaccination and for category III immediate vaccination and administration of rabies immunoglobulin (RIG) are recommended.^13^ After exposure to a dog bite, the following steps should be taken.

### Step 1: Wound care

Recommended first aid includes washing and flushing bite wounds with soap water, detergent, povidone-iodine and other viricidal solutions. Primary wound closure may be deferred except for facial bite wounds which may be closed primarily after thorough debridement and infiltration with RIG.^13,14^ Laceration injuries can be closed primarily, but avulsion injuries may benefit from delayed closure. Injuries with significant tissue loss may require a local flap, composite grafts or, sometimes, vascularised flaps. Antibiotics to cover suspected organisms and tetanus toxoids are routinely given, whilst some cases may require reconstructive surgery.^12,15^ Studies have shown that there is no difference in the wound infection rate (8.3%) between primary suturing and non-suturing group, but the cosmetic outcome of the primarily sutured wounds was found to be significantly better.^16^

### Step 2: Vaccination

The decision to initiate vaccination or not should be guided by the risk factors for rabies, which are as follows:^13^

1. Location (country) of exposure to dog bite as vaccination may not be needed in a rabies-free country
2. Severity of exposure: category II and III typically require post-exposure prophylaxis
3. Clinical behaviour of the dog, if available for observation
4. Vaccination status of the dog (animal), if available.

The decision to initiate anti-rabies vaccine should not be delayed so that post-exposure vaccination can be started whilst the dog is being observed. The World Health Organization (WHO) recommended schedules for pre-exposure and post-exposure vaccination can be administered either via intramuscular or intradermal route. The intramuscular route is universally recommended whilst the intradermal route is recommended for resource-constrained settings. The intradermal route has the advantage of reducing the number of visits for pre-exposure prophylaxis to one. The reduced costs also increase the likelihood of the patient completing the post-exposure prophylaxis. The intradermal route is not recommended for pre-exposure prophylaxis in immune-compromised patients or those taking chloroquine for treatment or prophylaxis of malaria.^13,14^

Whilst most countries use a five-dose schedule, many have now adopted the WHO four-dose regimen. Depending on the vaccine type and schedule adopted, the post-exposure prophylaxis schedule prescribes intramuscular doses of 1 millilitre (mL) or 0.5 mL given as four to five doses of a cell-derived rabies vaccine given on day 0, 3, 7 and 14 post-exposure. A fifth dose on day 28 is still recommended, especially in immune-compromised patients.^13,14^ The series need not be restarted if doses are not given exactly to schedule.^13,14^ For patients who have previously undergone pre-exposure prophylaxis or completed post-exposure treatment with cell-derived rabies vaccine, two intramuscular doses of a cell-derived vaccine are sufficient and no RIG is necessary.^13,14^

### Step 3: Rabies immunoglobulin

Rabies immunoglobulin provides passive antibodies at the site of exposure and is recommended for category III injuries. It is given immediately or within 7 days of the first vaccine dose before the patient develops an active immune response. The recommended dose is 20 IU/kg (international unit per kilogram) given in and around the wound.^2^

Complications of dog bite injuries are rabies infection, wound infection and tissue loss leading to reconstructive surgeries.^12^ In the United States, more than 31 000 patients required reconstructive surgery because of dog bite injuries in 2006.^17^ Dog bite repairs were amongst the top five reconstructive surgeries performed by plastic surgeons; it exceeds head and neck and lower extremity reconstructions.^18^ Plastic surgeons interact with a small fraction of patients with dog bite injuries and often only with severe cases.^15^ The emergency care physicians, paediatricians, primary care physicians and the parents are the vital front line in treatment, education and prevention of dog bite injuries.^18^

Data on the prevalence of dog bite injuries in South Africa are lacking. However, studies in other countries show varying incidences ranging from 500 000 to 1 million animal bites per year.^3^ A study in the United States showed an incidence of 18/1000 population.^19^ Studies conducted in Belgium on dog bite injuries in children revealed that 22/1000 children younger than 15 years were victims of dog bite annually.^20^ Another study of a new dog bite-related injury visits to the emergency departments in the United States was 333 687, a rate of 12.9 per 10 000 persons. This represents 914 new dog bite injuries requiring emergency department visits per day.^21^

A study conducted at a tertiary hospital in Nigeria^22^ showed that 81 patients out of 24 683 emergency department consultations presented with dog bite injuries, and the majority (55.6%) were children with male preponderance.^22^ Another study in Jos, Nigeria, showed 247 cases of dog bite injuries between May 2009 and June 2010, and 82.1% of the dogs involved had no anti-rabies vaccination.^23^ In South Africa, a study conducted between August 2007 and September 2011 in Ngwelezane Hospital in KwaZulu-Natal showed a total of 821 patients presented to the hospital with dog bite injuries.^24^ Of these, 485 were male patients (55.8%). The most common age group was between 6 and 10 years. Younger male individuals were bitten more commonly than younger female individuals.^24^

More attention and research need to be devoted to the study of prevalence and the profile of patients presenting with dog bite injuries in South Africa, as it remains a public health problem. Knowing the prevalence will help policy-makers understand the magnitude of dog bite injuries and consider the burden in terms of the financial cost of procuring vaccine, antibiotics and analgesia and the work hours lost because of dog bite injuries, and possibly mount or intensify awareness campaigns on dog bite injuries prevention measures.

Currently, no studies have been performed on the profile of dog bite injury patients or the prevalence of dog bite injuries in patients treated at Kimberley Hospital Complex (KHC), Northern Cape. Anecdotal evidence showed that dog bite injuries are still an important public health problem in this old city, which formed a good basis to conduct research on dog bite injuries in Kimberley to achieve the outlined objectives.

This study aimed to describe the profile of dog bite injuries in patients presenting for treatment at KHC’s emergency and gateway centres. Objectives were to determine the profile and prevalence of dog bite injuries in patients presenting at the hospital’s emergency and gateway centres as well as the compliance with mandatory notification of dog bite injuries by doctors at the hospital’s emergency and gateway centres.

## Methods

### Study design

This was a retrospective descriptive survey of all patients presenting at the emergency and gateway centres of KHC from 01 August 2015 to 31 July 2017.

### Setting

These centres are the main portal of entry for all patients to KHC, including patients with dog bite injuries. Kimberley Hospital Complex is the only tertiary hospital in the Northern Cape. The hospital has 650 beds and serves over 100 000 patients annually. It also serves as a primary and secondary health care centre for a population of just under one and a half million people.

### Study population

It was a hospital-based study where all the patients presenting at KHC’s emergency and gateway centres were considered and those with dog bite injuries were selected for inclusion. The doctors working in the emergency and gateway centre record information for all the patients seen in a patient registry and complete a notification form in compliance with the mandatory reporting of all dog bite cases. The notification forms are placed in a designated box and picked up by the Infectious Disease Surveillance and Control Unit of the hospital. All cases that were listed in the patient registry with the diagnosis of ‘dog bite’ at KHC’s emergency and gateway centres from 01 August 2015 to 31 July 2017 were included. All dog bite injuries by the South African K9 police dogs that were used for fighting crime were excluded as well as patients who presented with dog bite injuries outside the stipulated study period. Based on past experience, it was assumed that there would be approximately 500 cases with dog bite injuries presenting during the study period.

### Data collection

Data were recorded by the main author on a data collection sheet that was developed by the research team. Patients’ data were obtained from the patient registry where ‘dog bite’ was listed as the diagnosis. The patient registry at the emergency department and the gateway centres had the following demographic data: patient’s name, file number, address, date of birth, gender and contact number(s). Other information available in the registry were the time of patient assessment at the triage, attending doctor’s name and time of assessment, diagnosis and the treatment given. In the column for diagnosis and treatment, a brief description of the type and site of the dog bite and the treatment instituted by the doctor were documented. The patients’ registry is the most reliable document in terms of registering all patients seen at KHC.

Regarding the notification of dog bite injuries to the provincial Department of Health via the Infectious Disease Control and Surveillance Unit of the hospital, the data of the patients collected from the registry were compared to the data of the reported (notified) dog bite at the Infectious Disease Control and Surveillance Unit database. A detailed description of the category of dog bite injury and type of treatment given, if not documented in the patient registry, were obtained from the case notes of the patients.

A pilot study was conducted on the first five patients with dog bite injuries presenting at KHC’s emergency and gateway centres during the study period. The data for the pilot study were included in the main study, after extra information about the site of the dog bite was added.

### Data analysis

Collated data were transcribed into a Microsoft Excel sheet and analysed by the Department of Biostatistics, Faculty of Health Sciences of the University of the Free State, using statistical analysis software (SAS) Version 9.4 (Cary, NC: SAS Institute Inc; 2014). Results were summarised using descriptive statistics namely frequency and percentage for categorical data. The median and quartiles were used to describe the non-parametric numerical variables.

Prevalence was calculated by considering the total number of patients seen at KHC’s emergency and gateway centres during the study period as the denominator and the dog bite cases as the numerator.

### Ethical consideration

Ethical approval to conduct the study was obtained from the Health Sciences Research Ethics Committee, University of the Free State (HSREC 92/2017 [UFS-HSD2017/0983]) and the Provincial Health Research and Ethics Committee, Northern Cape Department of Health (NC_201710_001). Permission to access the patient registry was obtained from the KHC’s executives. No identifying information of the patients was captured, and data were handled confidentially.

## Results

During the study period, a total of 433 dog bite cases were identified out of the total number of patients seen at KHC emergency and gateway centres, resulting in a prevalence of 0.4%.

### Demographic information of patients with dog bite injuries

As seen in Table 1, most of the patients were male patients (62.4%) with the highest percentage of patients (19.6%) in the age group 10 to 19 years. The median age for cases with the most dog bite injuries was 26 years (range 11–44 years). The youngest case was less than a year old and the oldest 76 years.

**TABLE 1:** Demographic variables of dog bite injury cases.

Variable	*n*	%
**Gender**		
Male	270	62.4
Female	163	37.6
**Age range (years)**		
0–9	80	18.5
10–19	85	19.6
20–29	64	14.8
30–39	72	16.6
40–49	50	11.5
50–59	44	10.2
60–69	34	7.9
≥ 70	4	0.9

Table 2 shows the distribution of dog bite injuries by geographical location. Combined, more than 70% of dog bite patients came from Galeshewe (40.7%) and Kimberley (33.0%).

**TABLE 2:** Distribution of dog bit injuries according to geographical location.

Area	*n*	%
Galeshewe	176	40.7
Kimberley	143	33.0
Greenpoint	39	9.0
Richie	26	6.0
Modderrivier	6	1.4
Colville	5	1.2
Floors	4	0.9
Homestead	4	0.9
Platfontein	4	0.9
Other	26	6.0

**FIGURE 1:**
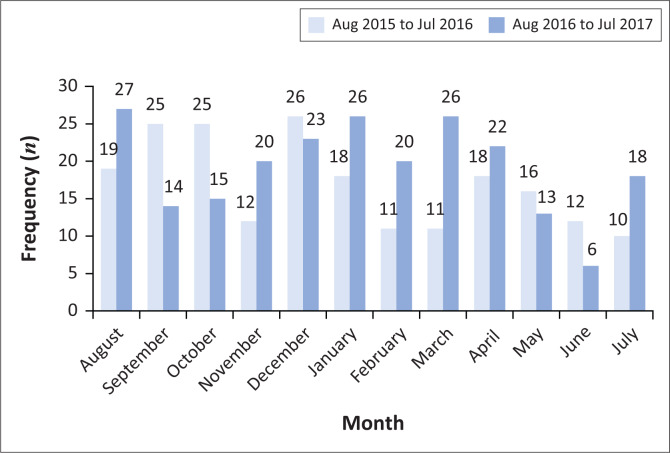
Monthly frequency of dog bite injuries during the study period.

During the study period, August 2015 had the highest percentage of dog bite injuries (6.2%), whilst June 2017 had the lowest percentage (1.4%) (Figure 1).

Table 3 shows that the most frequently injured anatomical sites were the lower limbs (51.7%) followed by the upper limbs (19.6%). In a high percentage (21.9%) of cases, the sites were not documented whilst 3.0% of injuries were at multiple anatomical sites.

**TABLE 3:** Distribution of dog bit injuries according to anatomical site.

Site of injury	*n*	%
Head and neck	6	1.4
Right upper limb	43	9.9
Left upper limb	42	9.7
Trunk	10	2.3
Right lower limb	105	24.3
Left lower limb	119	27.5
Multiple sites	13	3.0
Not documented	95	21.9

The dog bite injuries were categorised as follows: category I (1.2%, *n* = 5), category II (59.4%, *n* = 257) and category III (31.2%, *n* = 135). For 8.3% (*n* = 36) of the injuries, the category was not documented.

As shown in Table 4, anti-rabies vaccine was administered to 92.6% of the cases, 8.8% received anti-RIG, 94.2% cases received anti-tetanus toxoid, 90.8% cases were prescribed a course of antibiotic(s) and 93.3% were prescribed analgesics.

**TABLE 4:** Treatment administered or prescribed (*n* = 433).

Treatment	Administered		Not administered		Unknown	
	*n*	%	*n*	%	*n*	%
Anti-tetanus toxoid	408	94.2	8	1.9	17	3.9
Immunoglobulin	38	8.8	300	69.3	95	21.9
Rabies vaccine	401	92.6	12	2.8	20	4.6
Antibiotics	393	90.8	19	4.4	21	4.8
Analgesics	404	93.3	8	1.8	21	4.8

Out of the 135 category III exposure cases, only 38 (28.2%) received an anti-RIG as shown in Table 4.

Only 1.2% (*n* = 5) of all dogs were confirmed to have been fully vaccinated. The majority of cases were bitten by unvaccinated dogs (61.9%, *n* = 268), whilst 37.0% (*n* = 160) were not documented or had unknown vaccination status. Dogs unavailable for observation (stray) accounted for 83.1% (*n* = 360) of the dog bite injuries and only 1.6% (*n* = 7) were available for observation, whilst 15.2% (*n* = 66) were not documented.

### Prevalence of dog bite cases

During the study period, a total of 34 844 patients attended the KHC emergency centre, whilst 72 887 were seen at the hospital’s gateway centre, giving a total of 107 731 patients. This amounts to a prevalence of dog bite cases of 4/1000 patients.

### Compliance with mandatory notification of dog bite injuries

Out of the 433 cases of dog bite injuries treated at the centres, 207 (47.9%) were reported to the appropriate authority, whilst 225 (52.1%) were unreported by the treating doctors.

## Discussion

Dog bite injuries continue to be a preventable cause of injuries in all age groups and a significant public health danger in Kimberley city and its suburbs as seen from this study. Male patients (62.4%) were more affected compared to female patients, which is in keeping with other studies performed in South Africa and other parts of the world.^23,24,25^

In this study, there was a high incidence of dog bite injuries in certain geographical locations as compared to others; Galeshewe was the area with the most dog bites (*n* = 176, 40.7%) which is understood considering it as the suburb with the highest population in Kimberley. Central Kimberley added 33.0% to the burden, possibly because of poor control of domestic animals. Most of the bites were from stray dogs (83.1% of dogs unavailable for observation) suggesting that Galeshewe may have larger population of free-roaming dogs. It is interesting to note that Greenpoint (suburb), with a relatively small population, had a high burden of dog bite injuries (9.0%) relative to its population, possibly because of the presence of more informal settlements which are not favourable for keeping domestic animals. Literature has demonstrated a relationship between poverty, unemployment, poor housing and animal bites.^26^

In this study, the rate of dog bite injuries was higher in spring and summer. A likely explanation may be the increase in the number of children and adults outside during summer holidays and festive seasons. This finding is consistent with other studies performed in Ahaz County, South Western Iran,^27^ and in Jos City, Nigeria.^23^ A common age group for dog bite injuries was found to between 10 and 19 years (19.6%), which is consistent with the study performed in Ahaz,^27^ but in contrast to studies performed in India and Cape Town which showed ages between 0 and 5 years and less than 6 years, respectively.^25,28^ However, all the studies showed a significant number of cases amongst children aged below 10 years.^25,27,28^ Most of the bites were from unvaccinated dogs (61.9%) or those with unknown vaccination status (37.0%). Vaccinated dogs accounted for 1.2% of all bites – this is in contrast to a study performed in Mpumalanga which showed a vaccination status of 47% amongst dogs.^29^ A study carried out in Jos City, Nigeria, found that 93% of biting dogs were unvaccinated.^24^ The WHO advocates for a target of 70% rabies vaccination coverage for stray dogs.^30^

With regard to the anatomical site of injury, approximately half (51.7%) of the bites involved the lower limbs. Bites to the upper limbs constituted 19.6% of total dog bite injuries. Most studies showed preponderance of lower limbs.^24,25,27^ In children, head, neck, and upper extremity are the more affected anatomical sites.^1,26,31^

The WHO’s classification category II injuries (59.4%) were found to be the commonest exposure category consistent with a South African study,^25^ but contradicting a similar study performed in India which revealed the dominance of category III exposure.^28^ In this investigation, most cases (92.6%) received an anti-rabies vaccine and 94.2% a dose of tetanus toxoid. There were 31.2% cases of category III exposure who presented; only 28.2% of those received an anti-RIG. A possible explanation could be the lack of adequate knowledge on the indications for the administration of immunoglobulin and, to a lesser extent, stock-outs. There was a high rate of antibiotics usage (90.8%). The author could not distinguish between those prescribed prophylactically or those with clear indication(s) for use. There were no cases of human rabies disease recorded by the regular surveillance of rabies throughout the Northern Cape during the study period.

The prevalence of dog bite injuries in this study was found to be 0.4% (4/1000 people). This is not consistent with the study performed in Belgium and the United States which demonstrated higher prevalence rates (22 per 1000 and 15 per 1000, respectively).^18,19,20^ A possible explanation for the low prevalence rate at KHC could be as a result of the health-seeking behaviour of the individuals affected, with some cases, for example, seeking help at the private healthcare institutions and other not seeking medical help.

Of the 433 cases with dog bite injuries during the study period, less than half (47.9%) were notified to the appropriate authorities. Lack of awareness and the overwhelming number of patients attending the KHC emergency and gateway centres may be partly responsible for the low notification rate amongst doctors. Therefore, more awareness amongst doctors and other healthcare workers involved in treating dog bite injuries is required to step up the rate of notification to the public health authorities.

### Strengths and limitations of the study

There was no existing study on this topic in the Northern Cape and KHC in particular, so the study can be regarded as a benchmark study. Further research can be carried out for comparison. Some of the findings in this study were consistent with some national and international studies on this subject matter.

This study is dependent on the accuracy of the notes and completeness of the registers. Some data were not complete for some of the cases because of poor documentation by doctors and clerks. Some dog bite injury patients seek treatment at private hospitals and some do not come for treatment at all; this inadvertently reduced the number of cases of dog bite injuries treated at KHC and thereby affects the measured prevalence of dog bite injuries in the area.

### Recommendations

Based on the findings of this study, a multi-pronged approach to the prevention of dog bite injuries can be adopted in order to reduce the incidence and prevalence of dog bite injuries. Anti-RIG was significantly underutilised by health practitioners in the management of dog bite injuries. More awareness needs to be created amongst doctors and other healthcare workers, both in private and public sectors, on the need for notification of all dog bite injuries. This awareness can be enhanced via quality improvement cycles or by other measures that will foster prompt notification of all cases of dog (animal) bites. More effort should be targeted at the prevention of bites in children. There should be stringent measures to limit the number of free-roaming dogs.

## Conclusion

Dog bite injuries were found to be more common in children and adolescents. The prevalence appeared to decrease in adulthood with advancing age groups. Most bites were sustained from unvaccinated stray dogs. Category II exposure was the commonest. Dog bite injuries remain a public health problem in Kimberley. Barely half of the cases were notified to the appropriate authorities. Prevalence of dog bite injuries amongst patients presenting at KHC resulted in the low rate of 0.4%.
